# Correction: Im et al. Comparing Donor- and Acceptor-Originated Exciton Dynamics in Non-Fullerene Acceptor Blend Polymeric Systems. *Polymers* 2021, *13*, 1770

**DOI:** 10.3390/polym15224358

**Published:** 2023-11-08

**Authors:** Chan Im, Sang-Woong Kang, Jeong-Yoon Choi, Jongdeok An

**Affiliations:** Department of Chemistry, Konkuk University, 120 Neungdong-ro, Gwangjin-gu, Seoul 05029, Republic of Korea; tony22@konkuk.ac.kr (S.-W.K.); haru96@konkuk.ac.kr (J.-Y.C.); anhijuk@konkuk.ac.kr (J.A.)

There was an error in Equation (1) in the original publication [1]. The fill factor (FF) was written as a denominator erroneously although the FF must be a part of the numerator. Therefore, a correction has been made to Equation (1) in the Introduction section as follows.

(1) $$\mathrm{PCE}=\frac{P_{\mathrm{out}}}{P_{\mathrm{in}}}=\frac{J_{\mathrm{SC}}\times V_{\mathrm{OC}}\times \mathrm{FF}}{P_{\mathrm{in}}}$$

In addition, “And P_in_ and P_out_ in Equation (1) stand for the incident solar power and the converted output power, respectively.” is inserted into the first paragraph of Introduction as follows:

“Recently, non-fullerene type acceptors (NFA) have gathered remarkable attention owing to the rapid increase in their photovoltaic power conversion efficiency (PCE) [1–3]. One of the most important reasons for the impressive PCE improvement must be the extended absorption spectral range, which can provide higher short circuit current densities (J_SC_) as expressed by Equation (1) with additional solar cell parameters, e.g., open circuit voltage (V_OC_) and fill factor (FF). And P_in_ and P_out_ in Equation (1) stand for the incident solar power and the converted output power, respectively. Obviously, the absorption facility of an active layer must be a determining factor at the initial primary exciton (PE) forming stage of solar cell operation. However, for instance, charge transfer (CT) and charge carrier (CC) transport must be considered together because the photogenerated PEs must be converted to mobile CCs and extracted to external circuitry to be detected as J_SC_ [4]. Furthermore, most events occur in the solid states, such as CT and CC transport, and, therefore, J_SC_ must also strongly depend on their molecular morphology [5]. Nevertheless, this spectral extension is achieved by using noble NFA materials coupled with suitable polymers as a bulk heterojunction (BHJ) active layer. The benefits of NFA systems are clearly recognized by comparing with solar cells that comprise fullerene derivatives and, for instance, narrow bandgap polymers [6] or polythiophene derivatives [7].”

The authors apologize for any inconvenience caused and state that the scientific conclusions are unaffected. This correction was approved by the Academic Editor. The original publication has also been updated.
