# Supplementary material for: Comparing Donor- and Acceptor-Originated Exciton Dynamics in Non-Fullerene Acceptor Blend Polymeric Systems
Source: Polymers (Basel). 2021 May 28;13(11):1770. doi: 10.3390/polym13111770 (PMC8199303; doi:10.3390/polym13111770)
Supplement: Supplementary file 1 [file polymers-13-01770-s001.zip › polymers-1225462-supplementary.pdf]

polymers

Supplementary Materials

Comparing Donor- and Acceptor-Originated Exciton Dynamics in Non-Fullerene Acceptor Blend Polymeric Systems

Prof. C. Im*, S. W. Kang, J. Y. Choi, and J. An

Department of Chemistry, Konkuk University, 120 Neungdong-ro, Gwangjin-gu, Seoul 05029, Korea

E-mail: chanim@konkuk.ac.kr

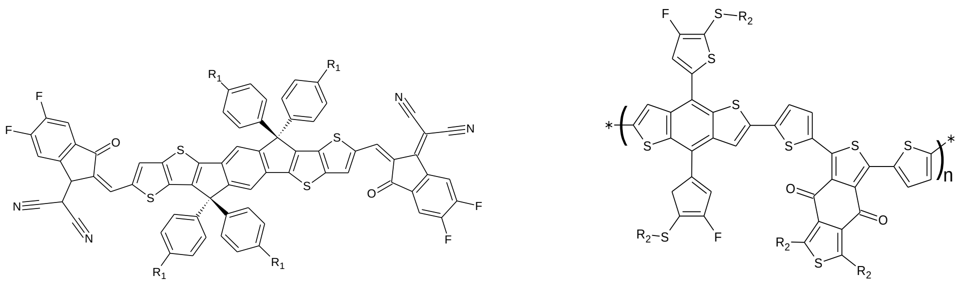

(A) ITIC-4F (B) PBDB-T-2F

Figure S1. Chemical structure of (A) ITIC-4F (**I**) and (B) PBDB-T-2F (**P**). (R_1 = 2-ethylhexyl, R_2 = hexyl).

PBDB-T and ITIC[4] stand for poly[(2,6-(4,8-bis(5-(2-ethylhexyl)thiophen-2-yl)-benzo[1,2-b:4,5-b']dithiophene))-alt-(5,5-(1',3'-di-2-thienyl-5',7'-bis(2-ethylhexyl)benzo[1',2'-c:4',5'-c']dithiophene-4,8-dione))] and 3,9-bis(2-methylene-(3-(1,1-dicyanomethylene)-indanone))-5,5,11,11-tetrakis(4-hexylphenyl)-dithieno[2,3-d:2',3'-d']-s-indaceno[1,2-b:5,6-b']dithiophene, respectively. ITIC-4F and PBDB-T-2F stand for their corresponding fluorinated derivatives.[6]

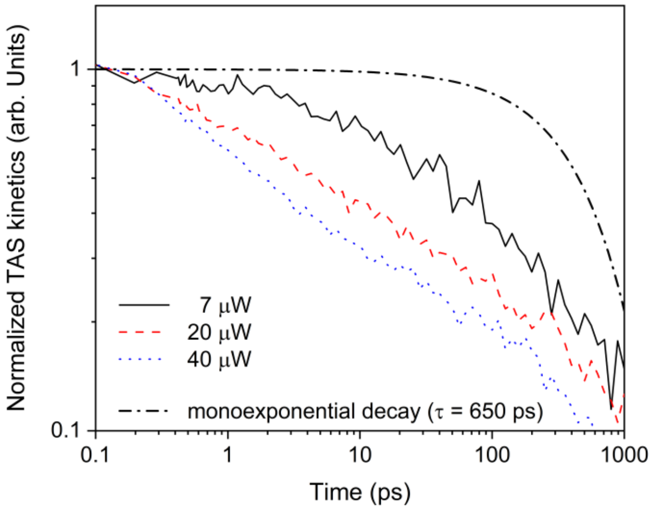

Figure S2. Excitation intensity dependent TAS kinetics of a **P** solution (λ_{exc} = 575 nm). Those normalized kinetic traces were extracted at the wavelength of ~1120 nm. The dash-dot line is the monoexponential decay curve calculated with a spectroscopic lifetime of 650 ps.

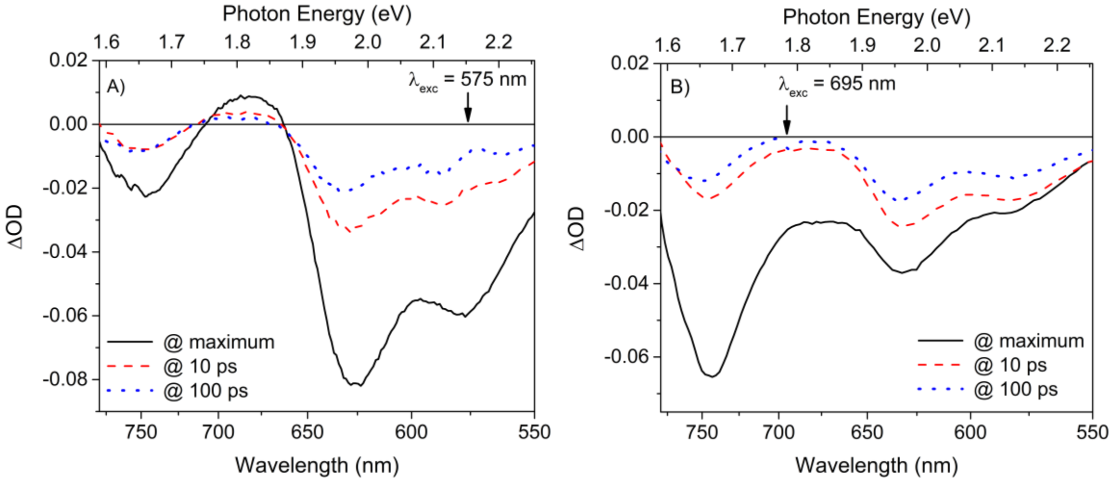

Figure S3. ΔOD spectra of blend film with time delays at approximately 0 ps (solid line), 10 ps (dashed line), and 100 ps (dotted line) excited at A) 575 nm and B) 695 nm.

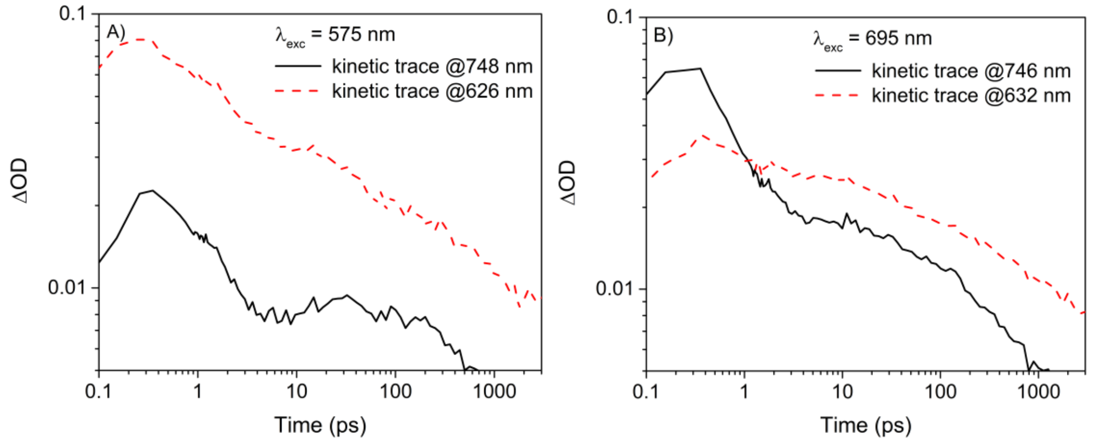

Figure S4. Kinetic traces extracted at various wavelengths of the visible wavelength range TAS spectra of blend films excited at A) 575 nm and B) 695 nm.

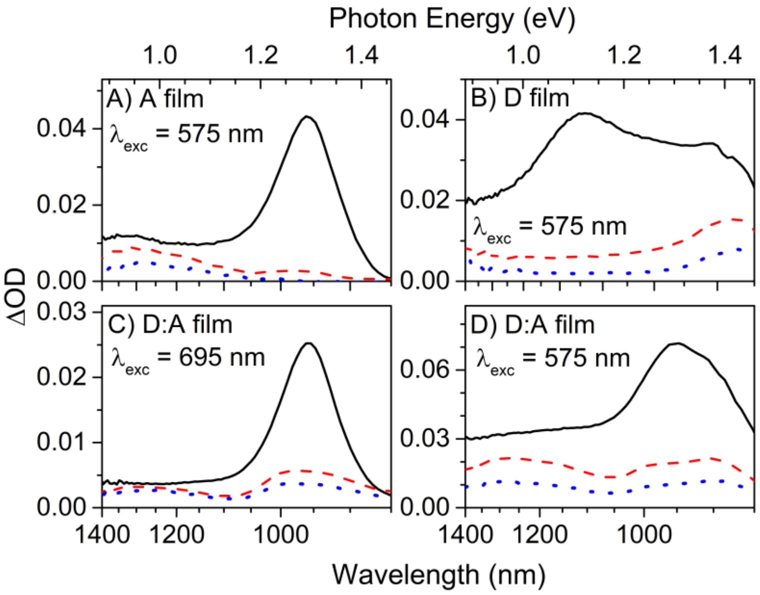

Figure S5. TA spectra of the A) ITIC-4F pristine film, B) PBDB-T-2F pristine film, and C) and D) their blend film. These spectra were extracted at time delays of about 0 ps (solid lined), 10 ps (dashed line), and 100 ps (dotted line). The spectra in D) corresponds to an excitation wavelength of 695 nm, while A), B), and C) correspond to an excitation wavelength of 575 nm.

polymers

Table S1. TAS decay time constants of **P** and **I** solution samples.

	λ_{decay} (nm)	FWHM (fs) of IRF	A_1	τ_1	A_2	τ_2	A_3	τ_3
PBDB-T-2F (**P** solution)	1123	274	3.56E-3	1.40 ns	8.51E-3	1.16 ps	6.00E-3	27.0 ps
ITIC-4F (**I** solution)	947	113	9.61E-3	789 fs	3.54E-3	45.5 ps	**1.18E-2**	**392 ps**

The listed spectroscopic lifetimes were estimated by fitting procedures according to the equation (4) shown in the manuscript. The λ_{decay} stands for the wavelength where the kinetic trace excited at the wavelength of 575 nm was extracted for the fitting procedure. The bold font marked lifetime of I was the dominant decaying component of the PE(I) solution and therefore, that was chosen to analyze exciton dynamics of the system further and listed in Table 2 of the manuscript.
